# Influence of sea ice concentration, sex and chick age on foraging flexibility and success in an Arctic seabird

**DOI:** 10.1093/conphys/coae057

**Published:** 2024-09-05

**Authors:** Alyssa Eby, Allison Patterson, Shannon Whelan, Kyle H Elliott, H Grant Gilchrist, Oliver P Love

**Affiliations:** Department of Integrative Biology, University of Windsor, 401 Sunset Avenue, Windsor, ON, N9B 3P4, Canada; Department of Natural Resource Sciences, McGill University, 21111 Lakeshore Road, Ste Anne-de-Bellevue, Quebec, H9X 3V9, Canada; Department of Natural Resource Sciences, McGill University, 21111 Lakeshore Road, Ste Anne-de-Bellevue, Quebec, H9X 3V9, Canada; Department of Natural Resource Sciences, McGill University, 21111 Lakeshore Road, Ste Anne-de-Bellevue, Quebec, H9X 3V9, Canada; Environment and Climate Change Canada, National Wildlife Research Centre, 1125 Colonel By Drive, Raven Road, Ottawa, ON, K1A OH3, Canada; Department of Integrative Biology, University of Windsor, 401 Sunset Avenue, Windsor, ON, N9B 3P4, Canada

**Keywords:** Climate change, diving behaviour, environmental variability, foraging behaviour, nutritional biomarkers, thick-billed murre

## Abstract

Declining sea ice and increased variability in sea ice dynamics are altering Arctic marine food webs. Changes in sea ice dynamics and prey availability are likely to impact pagophilic (ice-dependent and ice-associated) species, such as thick-billed murres (*Uria lomvia*), through changes in foraging behaviour and foraging success. At the same time, extrinsic factors, such as chick demand, and intrinsic factors, such as sex, are also likely to influence foraging behaviour and foraging success of adult murres. Here, we use 3 years of data (2017–2019) to examine the impacts of environmental conditions (sea ice concentration and sea surface temperature), sex and chick age (as a proxy for chick demand) on foraging and diving behaviour (measured via biologgers), energy expenditure (estimated from activity budgets) and foraging success (measured via nutritional biomarkers) of thick-billed murres during the incubation and chick-rearing stages at Coats Island, Nunavut. Murres only exhibited foraging flexibility to environmental conditions during incubation, which is also the only stage when ice was present. When more ice was present, foraging effort increased, murres foraged farther and made deeper dives, where murres making deeper dives had higher foraging success (greater relative change in mass). During incubation, murre behaviour was also influenced by sex of the individual, where males made more and shorter trips and more dives. During chick-rearing, murre behaviour was influenced primarily by the sex of the individual and chick age. Males made shallower dives and fewer dive bouts per day, and more dives. Birds made longer, deeper dives as chicks aged, likely representing increased intra-specific competition for prey throughout the season. Our results suggest variation in sea ice concentration does impact foraging success of murres; however, sex-specific foraging strategies may help buffer colony breeding success from variability in sea ice concentration.

## Teaser

Thick-billed murres from a low Arctic colony adjusted foraging behaviour in response to environmental conditions only during incubation. Whereas chick-rearing behaviour and foraging success were impacted by chick age and sex, reflecting the high costs of chick demand and sex-specific differences in parental care.

## Introduction

Climate change in Arctic regions is leading to a reduction in Arctic sea ice extent and greater inter-annual variability in sea ice dynamics, ultimately impacting Arctic marine food webs and prey availability, which poses a risk to Arctic breeding seabirds, such as thick-billed murres (*Uria lomvia*) (Macias-Fauria and Post, 2018; Mioduszewski *et al.*, 2019). Currently, thick-billed murre populations are rapidly declining in the northeastern Atlantic, coinciding with warming trends (Descamps and Strøm, 2021). Changes in extrinsic factors, especially environmental conditions, can indirectly impact fitness-related behaviours of seabirds, such as migration and nesting, through their effects on prey availability and foraging behaviour (Grémillet and Boulinier, 2009). For example, during the non-breeding season Atlantic puffins (*Fratercula arctica*) breeding on the Isle of May, Scotland did not adjust over-winter movements to environmental conditions, leading to lower reproductive success when environmental conditions were poor in the northeastern North Sea (St. John Glew *et al.*, 2019). In contrast, razorbills (*Alca torda*) from the same breeding colony adjusted their over-wintering foraging locations to match prey availability by migrating farther to more southerly regions of the North Sea, resulting in more stable reproductive success among years with contrasting environmental conditions (St. John Glew *et al.*, 2019). Biotic extrinsic factors, such as intra- and interspecific competition, or chick demand can also influence foraging behaviour. To offset the costs of chick demand, when adults must forage for themselves and their offspring, Manx shearwaters (*Puffinus puffinus*) adopt a bimodal foraging strategy during the chick-rearing stage, alternating between short trips to feed chicks and long trips to feed themselves, ultimately optimizing energy allocation (Shoji *et al.*, 2015).

In addition to being affected by multiple extrinsic factors, seabirds must also balance demands from numerous, often competing, intrinsic factors ranging from physiological function to the direct and indirect effects of phenotypic sex. Sex-specific behavioural differences have been observed in sexually size-dimorphic seabirds such as the wandering albatross (*Diomedea exulans*) (Weimerskirch *et al.*, 1997). However, sex-specific behavioural differences have also been observed in monomorphic seabirds such as little auks (*Alle alle*) and are thought to be a result of carry over effects associated with the intrinsic demands of reproduction (e.g. egg production in females; Welcker *et al.*, 2009). Notably in thick-billed murres, as males assume primary care of chicks following the breeding season they will typically feed on risk-averse, easy to catch prey with higher encounter rates, which allows them to feed both themselves and their chick (Elliott *et al.*, 2010). Risk averse prey items include diurnally migrating amphipods, which can lead to sex-specific diurnal foraging patterns, with females foraging during the day and males foraging at night (Elliott *et al.*, 2010). Birds experience both impacts of extrinsic and intrinsic factors on foraging behaviour in tandem, ultimately impacting foraging success and therefore both adult and offspring condition (Storey *et al.*, 2017). We thus expect that any constraints and trade-offs that seabirds face when trying to balance adult self-maintenance (e.g. nutritional state) and chick demand (extrinsic demand) will be more pronounced as environmental quality declines, necessitating adults to flexibly change foraging behaviour to maintain foraging success.

Despite strong conceptual predictions, quantifying how environmental variability and chick demand combine to impact the foraging behaviour and foraging success of adult free-ranging seabirds can be difficult. However, nutritional biomarkers measured from blood plasma, such as energetic metabolites and energetic hormones, along with measures of body condition, such as mass, provide an effective tool for estimating the nutritional state and foraging success of seabirds (Madliger *et al.*, 2018; Morales *et al.*, 2020; Tarroux *et al.*, 2020; Whitehead and Dunphy, 2022). For instance, higher nutritional state and foraging success is indicated by greater mass and elevated circulating levels of plasma triglycerides (Dietz *et al.*, 2009; Gerson and Guglielmo, 2013) and lower levels of baseline corticosterone (bCORT; McEwen and Wingfield, 2003; Benowitz-Fredericks *et al.*, 2008), beta-hydroxybutyrate (B-OH; Cherel *et al.*, 1988; Guglielmo *et al.*, 2005; Anteau and Afton, 2008) and non-esterified fatty acids (NEFA; Jenni-Eiermann and Jenni, 1994; Williams and Buck, 2010). As nutritional biomarkers have different turnover rates, it is therefore important to consider a suite of biomarkers for assessing nutritional state (Morales *et al.*, 2020; Eby *et al.*, 2023; Jackson *et al.*, 2023). Taking an integrative approach by combining behaviour, physiology and reproductive demand should improve our ability to understand how environmental change will impact the way in which these factors integrate to ultimately impact fitness. This understanding should in turn allow us to better predict how complex and high rates of environmental change are affecting demographic processes in species, such as thick-billed murres. Nutritional biomarkers thus can provide a mechanistic link between the impacts of climate change on foraging ecology and individual nutritional state, and therefore can be used a conservation tool to monitor population health (Eby *et al.*, 2023).

Here, we investigated how extrinsic demands, environmental variability and chick demand, and intrinsic factors, adult sex, interact to affect foraging flexibility and foraging success of thick-billed murres breeding in an Arctic region facing climate change. Specifically, we used a multi-year dataset to (i) investigate how environmental variability influenced foraging flexibility, i.e. the ability to adjust foraging behaviour (measured via biologgers) and foraging success (measured via nutritional biomarkers) during the incubation and chick-rearing breeding stages and (ii) assess the impact of chick age (as a proxy for chick demand) and adult sex on foraging flexibility and success. We hypothesized that both foraging and diving behaviour, and therefore subsequent foraging success, would vary in response to environmental conditions: murres should increase their search effort in warmer, less icy conditions (reduced prey availability) resulting in longer trips, as well as increase the amount of time and effort spent in foraging dives, leading to lower foraging success and negative impacts on parental nutritional state. As murres at our focal colony have sex-specific foraging patterns, where males forage at night and females during the day, and consequently have sex-specific diets, we expected differences in foraging patterns by sex. We thus hypothesized that females, which forage during the day, would make deeper dives and have higher foraging success, as they have greater access to high energy pelagic prey. We then hypothesized that murres would adjust foraging and diving behaviour in response to chick age: as chicks age (demand increases) murres should forage farther from the colony, as prey resources surrounding the colony become depleted, resulting in lower foraging success and negative impacts on parental nutritional state. Lastly, we hypothesized that both environmental conditions and chick age would interact with foraging behaviour to impact adult foraging success, where we predicted that foraging success and parental nutritional state would be lowest under warmer conditions and when chicks were oldest. By linking foraging flexibility and foraging success to environmental conditions and chick demand using an integrative behavioural–physiological approach our aim is to identify how extrinsic and intrinsic factors interact to impact an Arctic breeding seabird facing ever-increasing rates of climate change.

## Materials and Methods

### Study site: Coats Island, Nunavut

We conducted fieldwork at the Coats Island, Nunavut murre colony (62.95°N, 82.01°W) from 2017 to 2019. The Coats Island murre colony is located south of Salliq (Coral Harbour), Nunavut, in the northern Hudson Bay region and is an Environment and Climate Change Canada long-term monitoring site, with monitoring beginning in 1984 (Patterson *et al.*, 2024). Coats Island is a relatively small colony for the Canadian Arctic, with only 30 000 breeding pairs (Gaston *et al.*, 2012), where colony size is limited by available suitable nesting habitat on cliff ledges (Gaston *et al.*, 1993).

### Murre field sampling and GPS deployments

All field work was conducted under the University of Windsor Animal Utilization Project Proposal Permit (15-04), the McGill Animal Use Protocol (2015-7599) and Environment and Climate Change Canada Collection and Animal Care permits (NUN-SCI-14-11, EC-PN-14-017, EC-PN-15-017). During the incubation and chick-rearing stages, we caught adult murres at nest sites with a noose pole. After capture blood samples were drawn (1–2 ml using a 25-guage needle and 3 ml syringe) from the brachial or jugular vein and stored in a heparinized vacutainer. To effectively measure baseline physiology (see below), blood samples were drawn within 3 minutes of capturing. Blood samples were stored on ice (8 hours maximum) and whole blood was centrifuged at 10000 rpm for 5–10 minutes to separate plasma from red blood cells. Plasma was transferred into a cryovial and stored at −80°C until further processing. Additionally, we took a smear of red blood cells to molecularly sex birds (following Elliott *et al.*, 2010). As murres have sex-stereotyped foraging behaviour at Coats Island, any birds that were unable to be molecularly sexed (*n* = 28) were sexed based on behaviour (i.e. time at the nest; Elliott *et al.*, 2010). Murres were classified as females if they were on the nest consistently between 23 h30 and 3 h30 and murres were classified as males if they were on the nest consistently between 11 h30 and 15 h30 (previously shown to be 100% accurate when compared to DNA sexing, unpublished data, K. Elliott, person. Comm.).

Following blood sampling, murres were banded for individual identification. To quantify individual body size, we measured mass (g). Biologgers (GPS accelerometers, AXY-Trek™, Technosmart, 18 g, 1.9% of body mass) were then fitted to the dorsal feathers of adult murres (following Paredes *et al.*, 2005). Biologgers were set to record a GPS fix every 1 or 3 minutes, acceleration at 25 or 50 HZ in three axes, depth at 1 Hz and 0.1 m resolution and were left on for an average of 2 days. Murres were recaptured to retrieve biologgers, a second blood sample was taken and body mass was re-measured following removal of the biologger. In addition to biologger deployments, we monitored breeding plots daily to estimate lay date, hatch date and fledge date for each nest (following Gaston *et al.*, 2009) and to estimate chick age (our proxy for chick demand) at the time of GPS deployment, where chick age was averaged over the GPS deployment period.

### Environmental conditions

**Figure 1:**
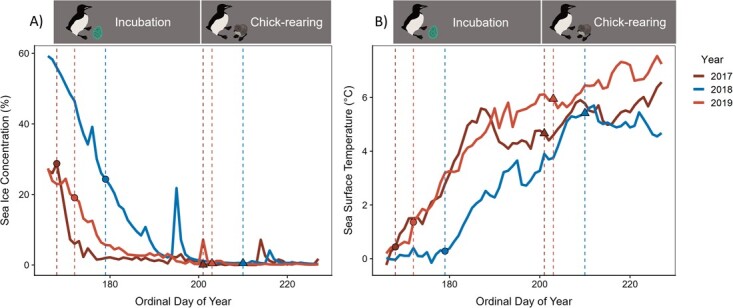
Sea ice concentration (%; A) and sea surface temperature (°C; B) throughout the thick-billed murre breeding season (15 June–15 August) for birds breeding at Coats Island, Nunavut, for each study year circles depict mean lay dates (2017 = 168/June 17th; 2018 = 179/June 28th; 2019 = 172/June 21st) and triangles depict mean hatch dates (2017 = 201/July 20th; 2018 = 210/July 29th; 2019 = 203/July 22^nd^), dashed lines highlight mean lay and hatch dates during incubation and chick-rearing stages. Low ice regime years (low sea ice extent, high SST) are shown in red and light red (2017 and 2019) and high ice regime years (high sea ice extent, low SST) are shown in blue (2018).

To investigate the impacts of environmental variability on foraging and diving behaviour between years, we measured sea ice concentration (%) and sea surface temperature (SST; °C) within a 130 km radius (the maximum foraging distance of murres at Coats Island, respective to study years) of the Coats Island colony for each year (2017–2019) during the breeding season—June 15th to August 15th (Fig. 1). Study years were previously categorized into sea ice regimes in Eby *et al.* (2023), with 2017 and 2019 being low ice regimes and 2018 being a high ice regime. Sea ice concentration and sea surface temperature were collected from the ‘global ocean Operational SST and Ice Analysis (OSTIA)’ remote sensing product from the Copernicus Marine Environment Monitoring Service (https://doi.org/10.48670/moi-00165). To examine foraging flexibility to environmental conditions during incubation, we averaged sea ice concentration over the biologger deployment period for each bird (see details below), as murres are thought to be ice-associated (Laidre *et al.*, 2008; Cusset *et al.*, 2019; LeBlanc *et al.*, 2019; Bonnet-Lebrun *et al.*, 2021, 2022). To examine foraging flexibility to environmental conditions during chick-rearing, we averaged sea surface temperature over the biologger deployment period, as there is little to no sea ice present during the chick-rearing stage.

### Foraging metrics, diving metrics and average daily energy expenditure

Biologger data were processed in *R* (version 4.21, R Core Team, 2022). We calculated wing-beat frequency, pitch, depth and distance from colony from biologger data to define behaviours (swimming, diving, flying and resting at the colony) using hidden Markov models with the “momentuHMM” package in R (McClintock and Michelot, 2018; following Patterson *et al.*, 2019). Once behaviours were classified, foraging trips were defined based on diving behaviour. If murres made more than one dive they were considered to be in a foraging trip. In addition, based on feeding-watch data (Elliott *et al*. unpubl. data) combined with biologger data, we observed murres making a single dive then delivering a prey item to their chick. Thus, to filter out dives associated with preening close to the colony, if murres made one dive that was greater than the median maximum dive depth of all years (18.1 m) they were also considered to be in a foraging trip.

For each trip, we calculated three foraging metrics, maximum distance travelled (km), total distance travelled (km) and trip duration (hours) and eight diving metrics were calculated maximum dive depth (m), mean dive depth (m), maximum dive duration (minutes), mean dive duration (minutes), number of dives, number of dive bouts (dive bouts were defined using a post-dive interval of 5 minutes), mean dives per dive bout and time diving (hours). We then used trip summaries to calculate foraging and diving metrics throughout the entire deployment. Deployment foraging metrics included maximum distance travelled (km), average daily distance travelled (km), mean trip distance (km), mean trip duration (hours) and number of trips per day. Diving deployment metrics included maximum dive depth (m), mean dive depth (m), maximum dive duration (min), mean dive duration (min), mean dives per dive bout, number of dives per day, number of dive bouts per day and time diving per day (hours). Classified behaviours were also used to calculate average daily energy expenditure (DEE; kJ/day) from the deployment period using the duration of each activity (T; hours) and estimates of energy associated with each activity (resting at the colony = 32 kJ, flying = 532.8 kJ, swimming = 100.8 kJ and diving = 97.2 kJ) from Elliott *et al.* (2013):


\begin{align*} &\mathrm{DEE}&\ \!\!\!\!\!\!\!\!\!=\!\! \left(\!\!\frac{32.0\!\ast\! T_\mathrm{{Colony}}\!+\!532.8\!\ast \!T_\mathrm{Flying}\!+\!100.8\!\ast \!T_\mathrm{Swimming}+\!97.2\!\ast \!T_\mathrm{Diving}}{\mathrm{Deployment}\ \mathrm{Duration}}\!\!\right) \!\ast \!24 \end{align*}

To simplify analyses, we ran principal component analysis (PCA) to reduce foraging and diving metrics to fewer relevant variables and when two factors were retained, they were varimax rotated. For foraging metrics, PCA generated two eigenvalues greater than one (2.85 and 1.50), collectively explaining 87.1% of the variation (Supplementary Table S1). For foraging rotated component one (fRC1), number of trips per day was strongly negatively loaded and mean trip distance and mean trip duration were strongly positively loaded (Supplementary Table S1). For foraging rotated component two (fRC2) maximum distance, average daily distance and mean trip distance were strongly positively loaded (Supplementary Table S1). For diving metrics, PCA generated two eigenvalues greater than one (3.56 and 2.15, respectively), collectively explaining 71.3% of the variance (Supplementary Table S2). For diving rotated component one (dRC1), maximum dive depth and mean dive depth were strongly negatively loaded, while maximum dive duration, mean dive duration, number of dive bouts per day and time diving per day were strongly positively loaded (Supplementary Table S2). On diving rotated component two (dRC2) mean number of dives per dive bout, number of dives per day and time diving per day were strongly positively loaded (Supplementary Table S2). To quantify the foraging area of murres, we pooled individuals for each breeding stage and study year and conducted kernel density analysis. We calculated the 95% and 50% utilization distributions from foraging locations (GPS locations associated with diving, locations associated with swimming, flying or resting at the colony were excluded) for each year and breeding stage with the adehabitatHR package, using a grid size of 800 metres and the ‘href’ ad hoc method to estimate smoothing parameters (Calenge, 2006). We calculated Bhattacharyya’s affinity index (BA) to measure spatial similarity between the 95% utilization distributions (overall foraging area) of each year within each breeding stage with the adehabitatHR package (Calenge, 2006), where the output ranges from 0 (no spatial similarity) to 1 (complete spatial similarity) (Fieberg and Kochanny, 2005).

### Nutritional state and foraging success

We measured mass, as well as, nutritional biomarkers (TRIG, bCORT, B-OH and NEFA) from plasma samples collected pre- and post-foraging to use post-foraging levels and relative change of mass/nutritional biomarkers (∆ = log(post-foraging levels)—log(pre-foraging levels)) to quantify nutritional state and foraging success of adult murres. For all study years, we ran a control within and across sample assay to calculate coefficients of intra- and inter-assay variation (Supplementary Table S3). To measure TRIG concentration (mmol/L), we used a commercially available assay kit (#TR0100-1KT; Sigma Aldrich, USA; previously validated by Williams *et al.,* 2007). To measure bCORT concentration (ng/mL), we used a commercial enzyme-linked immunoassay kit (EIA; Assay Designs, USA) to assay samples at a 1:40 dilution in triplicate (Hennin *et al.*, 2015). To measure B-OH concentration (mmol/L), we used a kinetic assay (SIGMA, Guglielmo *et al.*, 2002; previously validated by Lamarre *et al.,* 2017). To measure NEFA (mmol/L), we used a commercially available assay kit (NEFA HR2, Wako Diagnostics, USA; previously validated by Smith *et al.,* 2007 and Jeanniard du Dot *et al.,* 2009). See Supplementary File for detailed assay methods.

### Statistical analyses

#### Foraging and diving behaviour, energy expenditure and foraging success models

To assess the impact of environmental conditions (sea ice concentration) on foraging behaviour, average daily energy expenditure and foraging success of murres during incubation and assess the impact of environmental conditions (sea surface temperature) and chick age on foraging behaviour, average daily energy expenditure and foraging success of murres during chick-rearing we fitted linear mixed models (LMMs) using *lme4* (Bates *et al.*, 2015), where individual ID was fitted as a random effect to account for repeated samples of individuals (when sample size was sufficient) (see Supplementary File; see Table 1 for samples sizes). We fitted linear models when low number of repeated individuals did not allow for the use LMMs, where repeat individuals were removed to meet linear model assumptions. We included duration of biologger deployment in the models to account for variation in deployment length. For all foraging success models, time at the colony before the bird was sampled after returning from a foraging trip was included in the models to account for changes in nutritional biomarkers over time. We tested for multicollinearity of fixed effects in models by measuring variation inflation factors using the *Car* package (Fox and Weisberg, 2019). If the variance inflation factor or generalized variation inflation factor was respectively greater than 5 or 2.2 for a fixed effect or interaction between fixed effects, they were removed from the model. Models were ranked using Akaike Information Criterion corrected for small sample size (AICc) using the *MuMIn* package to determine the best ranked model (Burnham and Anderson, 2004; Barton, 2009). To test for model significance, we compared the top ranked model to the null model using an ANOVA (Supplementary Tables S4–S6). We visually inspected residuals versus fitted values plots to assess homogeneity of variance and quantile–quantile plots to assess normality for both fixed and random effects to check that the model assumptions were met. We then used restricted maximum likelihood estimation (REML) to re-fit models and obtained t-statistics using the *lmertest* package (Kuznetsova *et al.*, 2017). Lastly, we calculated marginal R^2^ (${r}_m^2$; proportion of variance in the model explained by the fixed effects) and conditional R^2^ (${r}_c^2$; proportion of variance in the model explained by fixed and random effects) for all models (Nakagawa *et al.*, 2017) using the *MuMIn* package.

**Table 1:** Sample size of thick-billed murre biologger deployments, post-foraging levels of nutritional biomarkers and relative change (∆) of nutritional biomarkers—mass, triglycerides (TRIG), baseline corticosterone (bCORT), beta-hydroxybutyrate (B-OH) and non-esterified fatty acids (NEFA) used in linear mixed models and linear models for study years (2017–2019) at Coats Island, Nunavut

	Breeding stage					
	Incubation 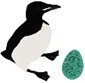			Chick-rearing 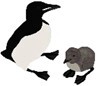		
	2017	2018	2019	2017	2018	2019
Deployments	13	75	82	51	46	70
post-Mass	8	71	81	46	29	37
post-TRIG	—	54	79	—	—	64
post-bCORT	—	53	71	—	—	64
post-B-OH	—	54	79	—	—	64
post-NEFA	—	54	70	—	—	69
∆ Mass	7	70	72	46	29	37
∆ TRIG	—	54	69	—	—	64
∆ bCORT	—	53	78	—	—	64
∆ B-OH	—	54	78	—	—	64
∆ NEFA	—	54	69	—	—	64

## Results

### Inter-annual variation in foraging area

**Figure 2:**
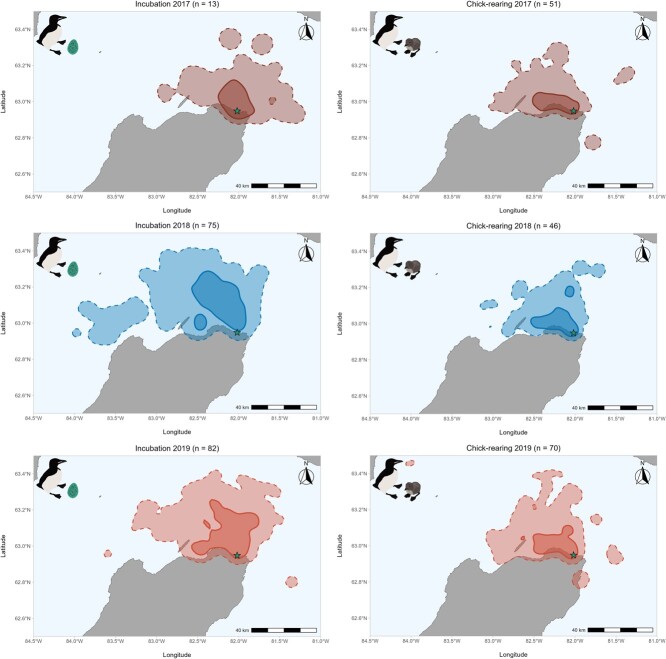
Foraging areas of thick-billed murres from Coats Island, Nunavut during the incubation (left panels) and chick-rearing stages (right panels) in 2017 (dark red, low sea ice regime), 2018 (blue, high sea ice regime) and 2019 (light red, low sea ice regime). Dashed lines represent the 95% utilization distribution (overall foraging area) and solid lines represent the 50% utilization distributions (core foraging area).

During incubation, murre foraging area (95% utilization distribution) was largest during 2018 (foraging area = 4026 km^2^; Fig. 2), where there was high spatial similarity with 2019 (BA = 0.84; foraging area = 3332 km^2^; Fig. 2) and moderate spatial similarity with 2017 (BA = 0.58; foraging area = 2495 km^2^; Fig. 2). In addition, there was moderate spatial similarity between 2017 and 2019 foraging ranges (BA = 0.69). During chick-rearing there was high spatial similarity among years (BA 2017–2018 = 0.81; BA 2017–2019 = 0.86; BA 2018–2019 = 0.83), with 2019 having the largest foraging area (2322 km^2^; Fig. 2), followed by 2017 (1764 km^2^: Fig. 2) and 2018 (1687 km^2^; Fig. 2).

### Environmental conditions

#### Incubation

**Figure 3:**
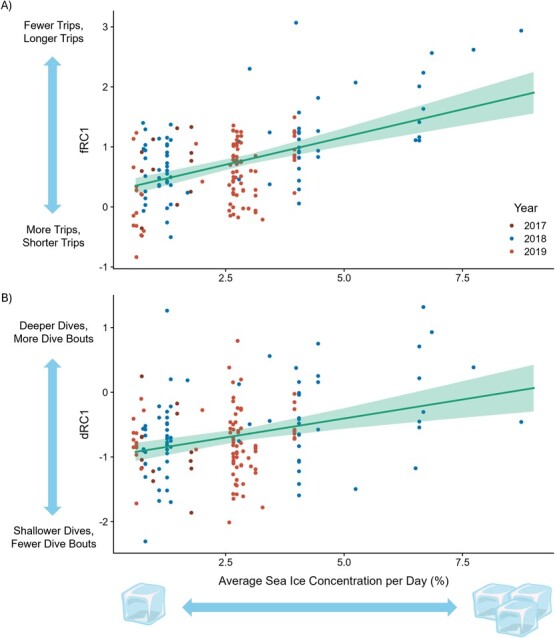
Relationship between average sea ice concentration per day (%) and foraging behaviour, fRC1 (A; mean trip distance, mean trip duration and number of trips per day) and dRC1 (B; maximum dive depth, maximum dive duration, mean dive depth, mean dive duration, number of dive bouts per day and time diving per day) during incubation at Coats Island, Nunavut in 2017 (low ice regime; dark red), 2018 (high ice regime; blue) and 2019 (low ice regime; light red). Turquoise line represents the marginal effect of average sea ice concentration per day on fRC1 and dRC1 and turquoise shading represents the 95% confidence interval.

When sea ice concentration was high, murres made fewer and longer trips (higher fRC1 scores, *P* < 0.001; Supplementary Table S7; Fig. 3), foraged more distantly (higher fRC2 scores, *P* < 0.001; Supplementary Table S7) and made deeper, longer dives and more dive bouts per day (higher dRC1 scores; *P* < 0.001; Supplementary Table S7; Fig. 3). We found an interaction between sea ice concentration and diving behaviour (dRC2) on relative change in mass (*P* = 0.04, Supplementary Table S10), where murres had greater relative change in mass, i.e. better nutritional state, when making deeper, longer dives and more dive bouts per day at higher sea ice concentrations. We also found that murres had lower relative change in B-OH, i.e. better nutritional state, when sea ice concentration was higher (*P* = 0.03, Supplementary Table S11).

#### Chick-rearing

In contrast to incubation, environmental conditions (sea surface temperature) did not influence foraging and diving behaviour during chick-rearing (Supplementary Table S13 and S14). Despite having only one year of physiological data for chick-rearing (2019; Table 1), we did find that sea surface temperature influenced foraging success and nutritional state of murres. Murres had higher foraging success and better nutritional state when sea surface temperatures were lower—greater relative change in mass (*P* = 0.04; Supplementary Table S15), greater post-foraging TRIG (*P* = 0.02; Supplementary Table S16), greater relative change in TRIG (*P* = 0.003; Supplementary Table S16) and lower post-foraging B-OH (*P* = 0.002; Supplementary Table S18).

### Sex

#### Incubation

Males made more trips and shorter trips (lower fRC1 scores; *P* = 0.02; Supplementary Table S7) and more dives (higher dRC2 scores; *P* = 0.002; Supplementary Table S8) than females. We also observed that males were in poorer nutritional state (lower post-foraging TRIG, *P* = 0.003; Supplementary Table S9) than females.

#### Chick-rearing

Males made shallower, shorter dives and fewer dive bouts per day (lower dRC1 scores; *P* = 0.03; Supplementary Table S13) and more dives (higher dRC2 scores; *P* < 0.001; Supplementary Table S14) than females. Sex also interacted with diving behaviour to influence nutritional state of murres, females making deeper, longer dives and more dive bouts per day were in a better nutritional state (higher post-foraging mass, *P* = 0.04; Supplementary Table S15). Additionally, males making fewer dives (lower dRC2 scores) and females making more dives (higher dRC2 scores) were also in better nutritional state (lower post-foraging bCORT; *P* = 0.02; Supplementary Table S17). Finally, sex influenced nutritional state, with males having greater relative change in mass (*P* = 0.02, Supplementary Table S15), lower post-foraging TRIG (*P* = 0.001; Supplementary Table S16), lower relative change in NEFA (*P* = 0.02; Supplementary Table S18) and lower post-foraging NEFA (*P* = 0.01; Supplementary Table S19).

### Chick age

#### Chick-rearing

**Figure 4:**
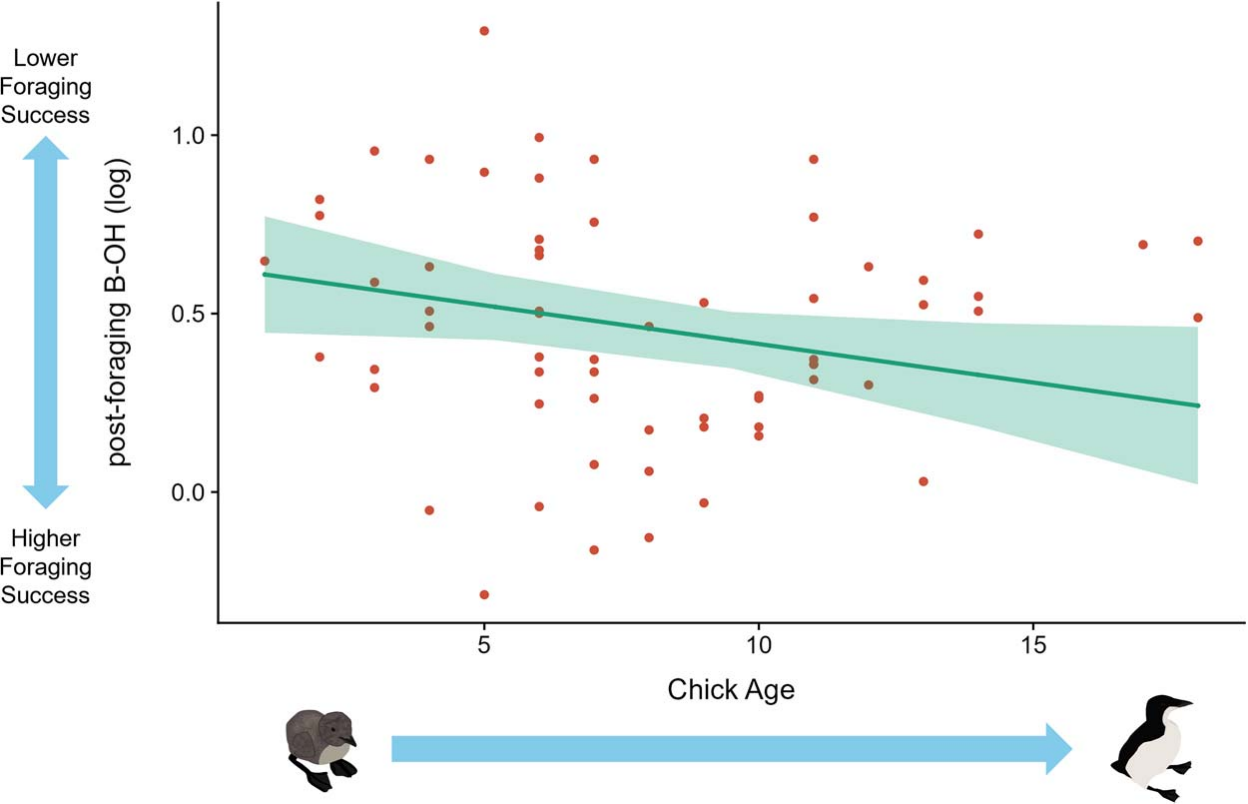
Relationship between chick age and adult foraging success—post-foraging beta-hydroxybutyrate levels (log-scaled) during chick-rearing at Coats Island, Nunavut in 2019 (low ice regime). Solid line represents the marginal effect of chick age on post-foraging B-OH levels and shading represents the 95% confidence interval.

Chick age influenced diving behaviour, where murres made deeper, longer dives and more dive bouts per day as chicks aged (higher dRC1 scores; *P* = 0.01; Supplementary Table S13). Diving behaviour (dRC1) and chick age interacted to influence adult nutritional state, murres attending older chicks made deeper and longer dives, had more dive bouts per day (higher dRC1 scores) and were in better nutritional state (lower relative change in B-OH, *P* = 0.03, Supplementary Table S18). Chick age also influenced nutritional state and foraging success, where post-foraging mass declined with chick age (*P* < 0.001; Supplementary Table S15) and post-foraging B-OH declined with chick age (*P* = 0.04; Supplementary Table S18, Fig. 4).

## Discussion

Murre foraging behaviour was influenced by environmental conditions only during incubation, which was also the only breeding stage when ice was present. Murres worked harder (flew farther and dove deeper) and had higher foraging success (lower relative change in beta-hydroxybutyrate) when sea ice concentration was higher. Additionally, at higher sea ice concentrations, murres making deeper, longer dives were in a better nutritional state (greater relative change in mass, Table 2). Presumably, murres were working harder to access rapidly dwindling cold-water prey when sea ice was still present and murres that were able to locate cold-water prey, such as Arctic cod, had greater energy payoffs. In contrast to incubation, foraging behaviour and success during chick-rearing was primarily determined by chick age and adult sex, representing the impact of chick demand and sex-specific diurnal patterns on foraging behaviour and foraging success at our focal colony (Table 2).

**Table 2:** Summary of linear mixed models and linear models investigating the impacts of environmental conditions (sea ice concentration and sea surface temperature), sex and chick age on the foraging behaviour, diving behaviour, foraging success and nutritional state of adult breeding thick-billed murres at Coats Island, Nunavut during the incubation and chick-rearing breeding stages

	Breeding stage	
Variables	Incubation 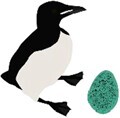	Chick-rearing 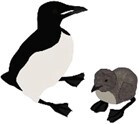
Environmental conditions 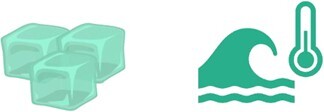	• Fewer and longer, more distant trips and deeper, longer dives and more dive bouts per day at high sea ice concentrations • At high sea ice concentrations deeper, longer dives resulted in better nutritional state	• No influence of sea surface temperature on foraging or diving behaviour • Higher foraging success and better nutritional state at low sea surface temperatures
Sex 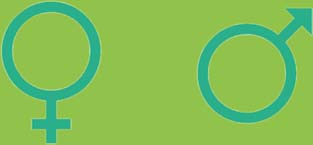	• Males made more trips and shorter trips and more dives than females • Males were in poorer nutritional state than females	• Males made shallower, shorter dives and fewer dive bouts per day and more dives than females • Females making deeper, longer dives and more dive bouts were in a better nutritional state
Chick age 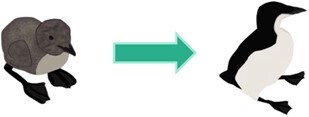		• Murres made deeper, longer dives and more dive bouts per day as chicks aged • As chicks aged, murres making deeper, longer dives and more dive bouts per were in a better nutritional state

### Environmental conditions

Murres exhibited foraging flexibility to environmental conditions. Birds made more and shorter trips, as well as shallower dives when sea ice concentration was lower. This did not match our initial predictions that murres would be make longer more distant foraging trips when sea ice concentration is lower as a result of lower prey availability. More trips and shorter trips when sea ice concentration was low resulted in smaller foraging ranges during low ice regimes in incubation. However, the smaller foraging range observed during the incubation of 2017 may be the result of a smaller sample size. Although murres exhibited foraging flexibility to environmental conditions, foraging success only varied across foraging strategies when sea ice concentration was high, where murres had greater foraging success (greater relative change in mass) when making deeper, longer dives and more dive bouts per day. Additionally, we found that murres making farther, longer trips had higher average daily energy expenditure, suggesting murres could be employing a high energy search strategy (Norberg, 2021; Patterson *et al.*, 2022).

Murres are considered an ice-associated species as they are thought to target sympagic Arctic cod (*Boreogadus saida*), cod foraging below the surface of sea ice (Cusset *et al.*, 2019; Pettitt-Wade *et al.*, 2021; Geoffroy *et al.*, 2023). Thick-billed murres are also considered cold-water specialists as they selectively forage in fjords with cooler waters and along the Marginal Ice Zone in Iceland (Bonnet-Lebrun *et al.*, 2021, 2022). As sea ice is dynamic, i.e. can move with ocean currents and wind, even when sea ice concentration is higher, sea ice may be more distantly located from the colony, leading to longer and more distant foraging trips, with murres using sea ice as a cue to locate Arctic cod (LeBlanc *et al.*, 2019; Wagner *et al.*, 2022). When sea ice concentration is low, i.e. there is little ice within their local foraging range, murres make shallower dives and shorter trips, which could represent a greater reliance on invertebrate prey, that is located in shallower waters in closer proximity to the colony (Brisson-Curadeau and Elliott, 2019).

Despite observing flexibility in foraging behaviour in response to sea ice concentration during incubation, murres did not exhibit foraging flexibility to environmental conditions (sea surface temperature) during chick-rearing. This likely reflects the costs associated with chick-provisioning, constraining murres to forage closer to the colony. Interestingly, environmental conditions did influence foraging success, where murres had higher foraging success (greater relative change in mass and triglycerides, greater post-foraging triglycerides and lower post-foraging beta-hydroxybutyrate) at lower sea surface temperatures. As sea surface temperature rises throughout chick-rearing this may then actually reflect increased intra-specific competition for prey throughout the breeding season, as prey becomes more depleted as the breeding season progresses leading to lower foraging success (Elliott *et al.*, 2009). A decline in foraging success throughout the season could also reflect the degree of parental investment via parents working hard to maintain chick-provisioning across the season, at a cost to themselves (see *Chick Age* section below).

### Sex

Irrespective of environmental conditions, we saw that females made fewer trips and longer trips, as well as fewer dives compared to males during incubation at the Coats Island colony. This supports previous work at this sub-polar colony which found that females targeted more pelagic prey sources (Woo *et al.*, 2008; Elliott *et al.*, 2010), which is thought to be a result of risk-partitioning between mates, where females forage during the day, feeding on risk-prone prey (pelagic species) as a result of greater light availability and males forage in the evening, feeding on risk-averse prey (invertebrates and benthic species; Elliott *et al.*, 2010). During chick-rearing we also found that females made fewer trips and longer trips, spending less time diving than males, again likely reflecting sex-specific differences in targeted prey. Previously collected diet data (stable isotopes and feeding watches) and diving data from the chick-rearing stage at this colony has shown that males typically feed on shallow water benthic species and amphipods in close proximity to the colony (Woo *et al.*, 2008; Elliott *et al.*, 2010; Elliott and Gaston, 2015; Brisson-Curadeau and Elliott, 2019). Similar to Coats Island, diurnal sex-specific colony attendance patterns are observed at both polar and subpolar thick-billed murre colonies (Huffeldt *et al.*, 2021). However, the pattern of sex-specific temporal segregation of colony attendance is not consistent across thick-billed murre colonies. Like Coats Island, females forage during the day and attend the colony at night at Kippaku and Kitsissut Avalliit in Greenland (Linnebjerg *et al.*, 2013, 2015; Huffeldt and Merkel, 2016; Huffeldt *et al.*, 2021). In contrast, males forage during the day and attend the colony at night at the Gannet Islands in the Atlantic (Jones *et al.*, 2002; Paredes *et al.*, 2006, 2008), Prince Leopold Island in the high Arctic (Elliott *et al.*, 2010) and St. Paul Island, St. George Island and Bogoslof Island in the Pacific (Roelke and Hunt, 1978; Young *et al.*, 2015). Interestingly, at Digges Sound, a subpolar colony in Nunavut, there is no temporal segregation of sexes, which is thought to be due to large colony size resulting in longer foraging trips and the breakdown of sex specific colony attendance patterns (Gaston and Noble, 1986; Hipfner *et al.*, 2006; Elliott *et al.*, 2009; Elliott *et al.*, 2009; Elliott *et al.*, 2010).

Regardless of temporal pattern by sex, at subpolar colonies where sex-specific temporal segregation occurs, vertical habitat segregation also occurs, where the sex foraging during the day makes deeper dives, whereas the sex foraging during the night makes shallower dives, as a result of diel vertical migration of prey and light availability (Paredes *et al.*, 2008; Elliott *et al.*, 2010; Linnebjerg *et al.*, 2015; Young *et al.*, 2015). Although vertical habitat segregation follows similar patterns across sub-polar colonies, this does not result in consistent patterns of diet specializations by sex across colonies (Young *et al.*, 2015; Huffeldt *et al.*, 2021). However, colony-specific differences may reflect context specific differences, such as bathymetry, colony size, environmental conditions and their interaction on preyscapes surrounding the colonies (Elliott *et al.*, 2010). Temporal segregation of sexes also occurs at polar thick-billed murre colonies, for example at Kippaku, where there is no difference in the light–dark cycle, however sexes exhibit reduced habitat segregation (Huffeldt *et al.*, 2021). This work suggests that temporal segregation results in differential habitat use by sexes at subpolar latitudes, as opposed to habitat preferences causing temporal segregation (Huffeldt *et al.*, 2021). Another possible driver of temporal segregation is social segregation, where females avoid males (Darden and Croft, 2008), or as a result of male defense of mates during the pre-laying stage (Paredes and Insley, 2010). Further investigation is needed into the drivers of temporal segregation patterns in thick-billed murres across their range.

Overall, we found that females had greater foraging success (higher post-foraging triglycerides) during incubation suggesting greater payoffs from pelagic prey (Elliott and Gaston, 2008). As females making deeper, longer dives were in better nutritional state, again this suggests higher pay-offs from pelagic prey items. We also found that males had lower post-foraging triglyceride levels, suggesting lower nutritional state. However, males also had lower relative change in non-esterified fatty acids and lower post-foraging levels of non-esterified fatty acids. Conflicting differences between these two physiological parameters may represent a difference in turnover rates of metabolites (Morales *et al.*, 2020; Jackson *et al.*, 2023). Additionally, differences in triglyceride levels and non-esterified fatty acids could be related to different energy pay-offs and energy demand of sex-specific foraging strategies. Triglycerides represent a combination of the energy pay-off from prey items and the amount of prey consumed (Dietz *et al.*, 2009), whereas, non-esterified fatty acids, represent energy demand (Jenni-Eiermann and Jenni, 1994; Williams and Buck, 2010). When non-esterified fatty acids are low, this means that energy payoffs are greater than energy demand. Males were found to make shallower, shorter dives and more dives than females, this foraging strategy is associated with foraging on amphipods, which have lower energy content compared to pelagic fish (Elliott and Gaston, 2008, 2015; Woo *et al.*, 2008; Elliott *et al.*, 2010; Brisson-Curadeau and Elliott, 2019). As males are consuming lower energy prey items, these results in lower triglycerides post-foraging. However, as males also have lower non-esterified fatty acids than females, this represents that the energy payoffs are greater than energy demand, therefore males are likely consuming enough lower energy prey to make up for energy demand.

### Chick age

Interestingly, we only observed an effect of chick age on diving behaviour, where murres made deeper, longer dives and more dives bouts per day as chick age increased. A change in dive depth could correspond to a shift in prey type or size of prey being targeted as chicks age (Elliott *et al.*, 2008; Elliott *et al.*, 2009). Diving behaviour and chick age influenced adult nutritional state—when chicks were older, murres making deeper, longer dives and more dive bouts per day were in better nutritional state (lower relative change in beta-hydroxybutyrate), suggesting that adults that are able to locate deeper, pelagic prey have higher pay-offs, compared to adults foraging on shallow-water invertebrates and amphipods (Cairns *et al.*, 1990; Elliott and Gaston, 2008; Elliott *et al.*, 2009).

Overall, we found that post-foraging mass declined with chick age. This is consistent with findings that murres undergo a programmed mass loss when chicks hatch, reflecting the high costs of chick-rearing, where adults adaptively lose mass to reduce the energetic costs (high flight costs) associated with foraging for themselves and chicks (Croll *et al.*, 1991; Gaston and Hipfner, 2006; Jacobs *et al.*, 2011). At the same time, we observed high post-foraging levels of beta-hydroxybutyrate when chicks were young, which is consistent with programmed adult mass loss, as beta-hydroxybutyrate is synthesized from free fatty acids to be used as fuel for tissues during periods of body mass loss (Williams *et al.*, 1999).

## Conclusions

Our observations of murres adjusting foraging behaviour in response to environmental conditions during incubation initially suggest that birds have an ability to cope with environmental change using foraging flexibility. However, by combining further information from physiological data on adult foraging success with foraging and diving behaviour across years of contrasting environmental conditions, we found that murres may still be detrimentally influenced by environmentally induced changes in prey availability. Our results suggest that differential habitat selection of sexes, as a result of temporal segregation due to differences in the light–dark cycle at a subpolar colony, may help buffer colony-level costs of environmental variability, where certain foraging strategies may do better in some years compared to others (Elliott *et al.*, 2010). Our findings highlight the use of nutritional biomarkers (energetic hormones and metabolites) as a means of assessing individual and population health. We therefore recommend the implementation of nutritional biomarkers into long-term monitoring programs, to provide a mechanistic link for understanding how species are affected by extrinsic factors, such as climate change. Integrating physiology into population monitoring and conservation programs should therefore lead to more informed and effective management decisions.

### Future directions

Continued work on this species should prioritize quantifying prey availability in the region to further understand how climate change is influencing prey abundance within this overall paradigm. Furthermore, future work should pair adult diet (measured via isotopes or fecal DNA analysis), prey capture events (measured via accelerometer data, e.g. wing-beat frequency), chick diet (measured via feeding watches) with chick-provisioning rates and adult foraging success (measured via nutritional biomarkers) to gain further insight into the trade-offs between self-provisioning and chick-provisioning in the face of climate change. Identifying these impacts and linkages are important as they will ultimately come together to impact breeding success. The expectation is that individuals in a long-lived species with low reproductive output will prioritize self-maintenance over chick provisioning in years of low food availability, to protect future reproductive investments at the cost of current chick investment (Ricklefs and Wikelski, 2002).

## Supplementary Material

Web_Material_coae057

## Data Availability

GPS tracking data are available in the Movebank Data Repository, https://doi.org/10.5441/001/1.341 (Eby et al., 2024).
