# Supplementary material for: Influence of sea ice concentration, sex and chick age on foraging flexibility and success in an Arctic seabird
Source: Conserv Physiol. 2024 Sep 5;12(1):coae057. doi: 10.1093/conphys/coae057 (PMC11381092; doi:10.1093/conphys/coae057)
Supplement: Web_Material_coae057 [file web_material_coae057.zip › CONPHYS-2023-011.R2_Supplementaryfile.pdf]

Laboratory Assays

Triglycerides (TRIG)

We diluted plasma (8 μL) with ultrapure water (8 μL) to run in duplicate with control plasma (Sigma-Aldrich, USA), a standard curve of the kit for the glycerol standard (Hennin et al. 2015), and glycerol reagent (240 μL), we then measured the solution with a spectrophotometer at 540 nm to measure free glycerol. We then added a triglyceride reagent (60 μl) to the solution and measured the solution with a spectrophotometer at 540 nm to measure total glycerol. Finally, to obtain TRIG concentration (mmol/L) we took the difference between free and total glycerol.

Baseline Corticosterone (bCORT)

To measure bCORT concentration (ng/mL) we placed 20 μL of plasma in a tube with 1 mL of distilled water and 5 mL of dichloromethane, vortexed the solution, and left the solution to separate for two hours. We then placed the dichloromethane phase into a scintillation vial, leaving the scintillation vial to evaporate in a fume hood. Once this evaporated, we rehydrated the samples with an assay buffer and vortexed the samples for 30 seconds. Finally, we assayed the samples at a 1:40 dilution in triplicate (Hennin et al. 2015).

Beta-hydroxybutyrate (B-OH)

We ran samples in duplicate, either adding B-OH dehydrogenase reagent (2 μL) and reagent buffer (2 μL) to plasma (11 μL) or to the standard (11 μL). The solution was then measured with a spectrophotometer at 492 nm to obtain B-OH concentrations.

Non-esterified fatty Acids (NEFA)

We ran samples in duplicate, adding 200 (μL) of reagent A and 100 (μL) of reagent B to plasma (5 μL), we then measured the solution with a spectrophotometer at 550 nm to obtain NEFA concentrations (mmol/L).

Statistical Analyses

Foraging and Diving Behaviour, Average Daily Energy Expenditure, and Foraging Success Models

During incubation, to assess the impact of environmental conditions and sex on foraging (fRC1 and fRC2) and diving behaviour (dRC1, dRC2) we fit LMMs or linear models with average sea ice concentration per day, and sex as fixed effects, year as a fixed effect to control for residual variation, and band as a random effect to account for repeated individuals, and fRC1, fRC2, dRC1, dRC2, respectively as the response variable. To assess the impact of environmental conditions, sex, foraging and diving behaviour on average daily energetic expenditure during incubation period we fit a LMM with average sea ice concentration per day, fRC1, fRC2, dRC1, dRC2, and sex as fixed effects. If sex or average sea ice concentration per day was significant in the foraging behaviour and diving behaviour models the interaction between sex or sea ice and the respective behaviour was included as a fixed effect in the average daily energy expenditure model, year was fitted as a fixed effect account to account for residual variation, band was fitted as a random effect to account for repeated individuals, and average daily energetic expenditure was fitted as the response variable. Lastly, during incubation to assess the impact of environmental conditions, sex, and foraging behaviour and diving behaviour on nutritional state and foraging success (post-foraging levels and relative change of mass, TRIG, bCORT, B-OH, and NEFA) we fit linear mixed models or linear models with average sea ice concentration per

day, fRC1, fRC2, dRC1, dRC2, and sex as fixed effects, if sex or average sea ice concentration per day was significant in the foraging behaviour and diving behaviour models the interaction between sex or sea ice concentration and the respective behaviour was included as a fixed effect, year was fitted as a fixed effect account to account for residual variation, band as a random effect to account for repeated individuals (LMMs only), and post-foraging mass (log-scaled), relative change in mass, post-foraging TRIG (log-scaled), relative change in TRIG, post-foraging bCORT (log-scaled), relative change in bCORT, post-foraging BOH (log-scaled), relative change in BOH, post-foraging NEFA (log-scaled), and relative change in NEFA respectively as response variables, where variables were log-scaled to meet model assumptions when necessary. For all foraging success models time at the colony before the bird was sampled after returning from a foraging trip (timebfbleed) was included in the model if it was significant, to account for changes in nutritional biomarkers over time. For all models duration of GPS deployment was included if it was significant to account variation in deployment lengths.

During the chick-rearing period, to assess the impact of environmental conditions, sex, and chick demand on foraging (fRC1 and fRC2) and diving behaviour (dRC1 and dRC2) we fit LMMs with average sea surface temperature per day, sex and chick age as fixed effects, year as a fixed effect to control for residual variation, and band as a random effect to account for repeated individuals, and fRC1, fRC2, dRC1, dRC2, respectively as the response variable. To assess the impact of environmental conditions, sex, foraging and diving behaviour, and chick demand on average daily energetic expenditure during the chick-rearing period we fit a LMM with average sea surface temperature per day, fRC1, fRC2, dRC1, dRC2, chick age, and sex as fixed effects, if sex, average sea surface temperature per day, or chick age was significant in the foraging behaviour and diving behaviour models the interaction between sex, sea surface temperature, or

chick age and the respective behaviour was included as a fixed effect, year was fitted as a fixed effect account to account for residual variation, band was fitted as a random effect to account for repeated individuals, and average daily energetic expenditure was fitted as the response variable. Lastly, during the chick-rearing period to assess the impact of environmental conditions, sex, foraging behaviour and diving behaviour, and chick demand on nutritional state and foraging success (post-foraging levels and relative change of mass, TRIG, bCORT, B-OH, and NEFA) we fit LMMs or linear models with average sea surface temperature per day, fRC1, fRC2, dRC1, dRC2, chick age, sex, and the interaction of chick age and sex as fixed effects. If sex, sea surface temperature, or chick age was significant in the foraging behaviour and diving behaviour models the interaction between sex, sea surface temperature, or chick age and the respective behaviour was included as a fixed effect. As we only had one year of data for physiological parameters during chick-rearing (2019), year was not included in the model, band was fitted as a random effect to account for repeated individuals (LMMs only), and post-foraging mass, relative change in mass, post-foraging TRIG (log-scaled), relative change in TRIG, post-foraging bCORT (log-scaled), relative change in bCORT, post-foraging BOH (log-scaled), relative change in BOH, post-foraging NEFA (log-scaled), and relative change in NEFA were fitted respectively as response variables, where variables were log-scaled to meet model assumptions when necessary. For all foraging success models time at the colony before the bird was sampled after returning from a foraging trip (timebfbleed) was included in the model if it was significant, to account for changes in nutritional biomarkers over time. For all models duration of GPS deployment was included if it was significant to account for variation in deployment lengths.

TABLES

Table S1. Factor loadings from principal components analysis on foraging metrics (maximum distance, average daily distance, mean trip distance, mean trip duration, and number of trips per day) from GPS deployments on thick-billed murres at Coats Island, Nunavut. Significant factor loadings are bolded.

	**fRC1**	**fRC2**
Maximum Distance	0.55	**0.73**
Average Daily Distance	-0.32	**0.90**
Mean Trip Distance	**0.76**	**0.60**
Mean Trip Duration	**0.93**	0.01
Number of Trips per Day	**-0.89**	0.10
Eigenvalue	2.85	1.50
Percentage of Variance Explaine	57.1	30.0

Table S2. Factor loadings from principal components analysis on diving metrics (maximum dive depth, maximum dive duration, mean dive depth, mean dive duration, mean dives per bout, number of dive bouts per day, number of dives per day, and time diving per day) from GPS deployments on thick-billed murres at Coats Island, Nunavut. Significant factor loadings are bolded.

	**dRC1**	**dRC2**
Maximum Dive Depth	**-0.89**	-0.04
Maximum Dive Duration	**0.85**	-0.08
Mean Dive Depth	**-0.86**	0.22
Mean Dive Duration	**0.62**	-0.30
Mean Dives per Dive Bout	-0.36	**0.75**
Number of Dive Bouts per Day	**0.62**	0.15
Number of Dives per Day	-0.02	**0.96**
Time Diving per Day	**0.60**	**0.72**
Eigenvalue	3.56	2.15
Percentage of Variance Explained	44.4	26.9

Table S3. Coefficients of intra- and inter- assay variation (%) for plasma triglyceride (TRIG), baseline corticosterone (bCORT), beta-hydroxybutyrate (B-OH), and non-esterified fatty acid (NEFA) assays on plasma samples from 2018 and 2019.

	**2018**	**2019**
TRIG		
Intra-assay variation	8.03	4.89
Inter-assay variation	20.56	23.34
bCORT		
Intra-assay variation	10.31	10.97
Inter-assay variation	11.93	16.52
B-OH		
Intra-assay variation	3.77	4.47
Inter-assay variation	5.23	4.52
NEFA		
Intra-assay variation	5.99	6.62
Inter-assay variation	4.30	6.64

Table S4. Model comparison of top-ranked and null linear mixed models and linear models for thick-billed murre foraging behaviour – fRC1 (mean trip distance, mean trip duration, and number of trips per day) and fRC2 (maximum distance, average daily distance, and mean trip distance), diving behaviour – dRC1 (maximum dive depth, maximum dive duration, mean dive depth, mean dive duration, number of dive bouts per day) and dRC2 (mean dives per bout, number of dive bouts per day, and time diving per day), and average daily energetic expenditure (DEE; log-scaled) during incubation and chick-rearing at Coats Island, Nunavut.

Model		N	Model Comparison			ANOVA	
			AICc	ΔAICc	w	χ2/F	p-value
Incubation							
main	fRC1 ~ AvgIceConc_day + Duration + Sex + Year + 1|Band	170	252.8	0.00	0.83		
null	fRC1 ~ 1|Band	170	333.8	81.0	0.00	91.8	**< 0.001**
main	fRC2 ~ AvgIceConc_day + Year + 1|Band	170	451.8	0.00	0.42		
null	fRC2 ~ 1|Band	170	480.5	28.7	0.00	35.1	**< 0.001**
main	dRC1 ~ AvgIceConc_day + 1|Band	170	290.4	0.00	0.15		
null	dRC1 ~ 1|Band	170	318.4	28.1	0.00	16.8	**< 0.001**
main	dRC2 ~ Sex + Year	159	422.9	0.00	0.22		
null	dRC2 ~ 1	159	429.1	6.18	0.01	4.20	**0.01**
main	logDEE ~ fRC2 + dRC1 + dRC2 + Year + 1|Band	170	-280.6	0.00	0.10		
null	logDEE ~ 1|Band	170	-134.7	146	0.00	2748	**< 0.001**
Chick-rearing							
main	fRC1 ~ Duration + 1|Band	167	358.1	0.00	0.16		
null	fRC1 ~ 1|Band	167	368.5	10.40	0.00	13.2	**< 0.001**
main	fRC2 ~ Sex + Year + Duration + 1|Band	167	436.7	0.00	0.12		
null	fRC2 ~ 1|Band	167	439.3	2.72	0.03	11.3	**0.02**
main	dRC1 ~ ChickAge + Sex + Year +Duration + 1|Band	167	392.9	0.00	0.20		
null	dRC1 ~ 1|Band	167	400.7	7.74	0.00	18.5	**0.002**
main	dRC2 ~ AvgSST_day + ChickAge + Sex + 1|Band	167	422.6	0.00	0.24		
null	dRC2 ~ 1|Band	167	463.2	40.60	0.00	45.0	**< 0.001**
main	DEE ~ fRC1 + fRC2 + dRC2 + Year + 1|Band	167	2187	0.00	0.10		
null	DEE ~ 1|Band	167	2492	304	0.00	315	**< 0.001**

Table S5. Model comparison of top-ranked and null linear mixed models and linear models for thick-billed murre nutritional state – post-foraging mass (post-Mass; log-scaled), post-foraging triglycerides (post-TRIG; log-scaled), post-foraging baseline corticosterone (post-bCORT; log-scaled), post-foraging beta-hydroxybutyrate (post-B-OH; log-scaled), and post-foraging non-esterified fatty acids (post-NEFA; log-scaled) and foraging success – relative change in mass (ΔMass), relative change in triglycerides (ΔTRIG), relative change in baseline corticosterone (ΔbCORT), relative change in beta-hydroxybutyrate (ΔB-OH), and relative change in non-esterified fatty acids (ΔNEFA) during incubation at Coats Island, Nunavut.

			**Model Comparison**			**ANOVA**	
	**Model**	**N**	**AICc**	**ΔAICc**	**w**	**χ2**	**p-value**
main	logpostMass ~ dRC2 + Year + TimebfSampling + 1|Band	160	-475.1	0.00	0.10		
null	logpostMass ~ 1|Band	160	-426.8	48.3	0.00	2259	**< 0.001**
main	ΔMass ~ fRC2 + dRC1*AvgIceConc_day + dRC2 + Year + Duration	149	-551.2	0.00	0.08		
null	ΔMass ~ 1	149	-500.6	50.7	0.00	10.2	**< 0.001**
main	logpostTRIG ~ fRC1 + dRC2 + Sex + Year + 1|Band	133	122.4	0.00	0.14		
null	logpostTRIG ~ 1|Band	133	155.1	32.6	0.00	41.2	**< 0.001**
main	ΔTRIG ~ fRC1 + fRC2 + dRC2	123	153.8	0.00	0.05		
null	ΔTRIG ~ 1	123	177.0	23.2	0.00	10.8	**0.001**
main	logpostCORT ~ AvgIceConc_day + fRC2 + dRC1	124	342.2	0.00	0.05		
null	logpostCORT ~ 1	124	346.9	4.64	0.00	3.73	**0.01**
main	ΔCORT ~ fRC2 + dRC2 + 1|Band	131	412.2	0.00	0.04		
null	ΔCORT ~ 1|Band	131	418.4	6.15	0.00	10.4	**0.01**
main	logpostBOH ~ fRC1 dRC1 + dRC2 + 1|Band	133	110.7	0.00	0.05		
null	logpostBOH ~ 1|Band	133	125.3	14.60	0.00	21.1	**< 0.001**
main	ΔBOH ~ AvgIceConc_day + dRC2 + Year + Duration + 1|Band	132	190.7	0.00	0.05		
null	ΔBOH ~ 1|Band	132	200.7	9.93	0.00	18.7	**< 0.001**
main	logpostNEFA ~ dRC1 + TimebfSampling	124	192.7	0.00	0.02		
null	logpostNEFA ~ 1	124	193.1	0.36	0.02	2.46	0.09
main	ΔNEFA ~ dRC2 + Year	123	280.8	0.00	0.01		
null	ΔNEFA ~ 1	123	281.8	1.10	0.01	2.7	0.08

Table S6. Model comparison of top-ranked and null linear mixed models and linear models for thick-billed murre nutritional state – post-foraging mass (post-Mass; log-scaled), post-foraging triglycerides (post-TRIG; log-scaled), post-foraging baseline corticosterone (post-bCORT; log-scaled), post-foraging beta-hydroxybutyrate (post-B-OH; log-scaled), and post-foraging non-esterified fatty acids (post-NEFA; log-scaled) and foraging success – relative change in mass (ΔMass), relative change in triglycerides (ΔTRIG), relative change in baseline corticosterone (ΔbCORT), relative change in beta-hydroxybutyrate (ΔB-OH), and relative change in non-esterified fatty acids (ΔNEFA) during chick-rearing at Coats Island, Nunavut.

			Model Comparison			ANOVA	
Model		N	AICc	ΔAICc	w	χ2	p-value
main	postMass ~ fRC2 + dRC1*Sex + dRC1 + ChickAge + TimebfBleed	112	1198	0.00	0.03		
null	postMass ~ 1	112	1215	17.1	0.00	5.18	**< 0.001**
main	ΔMass ~ AvgSST_day + fRC2 + dRC1 + Sex + Duration	112	-415.2	0.00	0.01		
null	ΔMass ~ 1	112	-406.6	8.63	0.00	4.05	**0.002**
main	logpostTRIG ~ AvgSST_day + fRC2 + Sex	64	88.66	0.00	0.04		
null	logpostTRIG ~ 1	64	110.7	22.0	0.00	11.4	**< 0.001**
main	ΔTRIG ~ AvgSST_day + fRC2 + dRC2 + Duration	64	80.00	0.00	0.04		
null	ΔTRIG ~ 1	64	96.44	16.4	0.00	7.30	**< 0.001**
main	logpostbCORT ~ fRC2 + dRC2*Sex	64	157.9	0.00	0.06		
null	logpostbCORT ~ 1	64	162.7	4.86	0.01	3.65	**0.01**
main	ΔbCORT ~ fRC2	64	194.9	0.00	0.01		
null	ΔbCORT ~ 1	64	196.0	1.04	0.01	3.21	0.08
main	logpostBOH ~ AvgSST_day + dRC1 + ChickAge + Sex	64	31.46	0.0	0.02		
null	logpostBOH ~ 1	64	38.66	7.20	0.00	4.33	**0.004**
main	ΔBOH ~ AvgSST_day + dRC1*ChickAge	64	73.91	0	0.03		
null	ΔBOH ~ 1	64	75.11	1.20	0.01	2.62	**0.04**
main	logpostNEFA ~ fRC1 + Sex + 1|Band	69	110.1	0.00	0.06		
null	logpostNEFA ~ 1|Band	69	117.1	6.99	0.00	11.6	**0.003**
main	ΔNEFA ~ Sex	64	117.0	0.00	0.05		
null	logΔNEFA ~ 1	64	120.4	0.01	0.01	5.66	0.02

Table S7. Summary of variation in thick-billed murre foraging behaviour – fRC1 (mean trip distance, mean trip duration, and number of trips per day) and fRC2 (maximum distance, average daily distance, and mean trip distance) and diving behaviour – dRC1 (maximum dive depth, maximum dive duration, mean dive depth, mean dive duration, number of dive bouts per day) and average daily energetic expenditure (DEE; log-scaled) from linear mixed models during incubation at Coats Island, Nunavut.

Model	Fixed Effects	Estimate ± SE	df	t	p	r_m^2	r_c^2
**fRC1 ~ AvgIceConc_day + Sex + Year + Duration + 1|Band**						0.41	0.61
	Intercept	0.15 ± 0.20	163	0.74	0.46		
	AvgIceConc_day	0.18 ± 0.03	163	6.82	**< 0.001**		
	Sex - M	-0.21 ± 0.09	154	-2.36	**0.02**		
	Year - 2018	0.02 ± 0.16	141	0.11	0.91		
	Year - 2019	-0.30 ± 0.16	153	-1.94	**0.05**		
	Duration	0.01 ± 0.00	161	3.19	**0.001**		
**fRC2 ~ AvgIceConc_day + Year + 1|Band**						0.16	0.85
	Intercept	-1.28 ± 0.25	105	-5.21	**< 0.001**		
	AvgIceConc_day	0.17 ± 0.04	89.0	4.07	**< 0.001**		
	Year - 2018	0.79 ± 0.25	53.4	3.16	**0.003**		
	Year - 2019	0.53 ± 0.26	72.0	2.07	**0.04**		
**dRC1 ~ AvgIceConc_day + 1|Band**						0.09	0.13
	Intercept	-0.99 ± 0.09	167	-11.60	**< 0.001**		
	AvgIceConc_day	0.12 ± 0.03	166	4.15	**< 0.001**		
**logDEE ~ fRC2 + dRC1 + dRC2 + Year + 1|Band**						0.61	0.77
	Intercept	7.77 ± 0.03	161	239	**< 0.001**		
	fRC2	0.13 ± 0.01	162	14.8	**< 0.001**		
	dRC1	0.03 ± 0.01	160	2.10	**0.04**		
	dRC2	0.02 ± 0.01	157	2.48	**0.01**		
	Year - 2018	-0.13 ± 0.03	148	-4.13	**< 0.001**		
	Year - 2019	-0.09 ± 0.03	159	-2.69	**0.01**		

Linear mixed model output includes estimates of fixed effects, standard error (SE), degrees of freedom (df), t-statistics (t), p-values (p), marginal R^2 (r_m^2; the proportion of variance in the model explained by the fixed effects) and conditional R^2 (r_c^2; the proportion of variance in the model explained by both fixed and random effects). Significant p-values in bold. See methods for full descriptions of models.

Table S8. Summary of variation in thick-billed murre diving behaviour – dRC2 (mean dives per bout, number of dive bouts per day, and time diving per day) from a linear model during incubation at Coats Island, Nunavut.

Model	Fixed Effects	Estimate ± SE	t	p	R^2_{adj}
**dRC2 ~ Sex + Year**					0.06
	Intercept	-0.99 ± 0.29	-3.40	**0.001**	
	Sex - M	0.53 ± 0.17	3.17	**0.002**	
	Year - 2018	0.34 ± 0.27	1.24	0.22	
	Year - 2019	0.56 ± 0.27	2.05	**0.04**	

Linear model output includes estimates of fixed effects, standard error (SE), t-statistics (t), p-values (p), and adjusted R^2 (R_{adj}^2; the proportion of variance in the model explained fixed effects). Significant p-values bolded. See methods for full descriptions of models.

Table S9. Summary of variation in thick-billed murre nutritional state – post-foraging mass (post-Mass; log-scaled), post-foraging triglycerides (post-TRIG; log-scaled), and foraging success – relative change in baseline corticosterone (ΔbCORT) from linear mixed models during incubation at Coats Island, Nunavut.

Model	Fixed Effects	Estimate ± SE	df	t	p	r_m^2	r_c^2
postMass							
**logpostMass ~ dRC2 + Year + timebfsampling + 1|Band**						0.23	0.9
	Intercept	6.80 ± 0.02	38.1	384	**< 0.001**		
	dRC2	0.01 ± 0.00	57.0	1.92	0.06		
	Year - 2018	0.10 ± 0.02	26.1	6.29	**< 0.001**		
	Year - 2019	0.12 ± 0.02	35.1	6.74	**< 0.001**		
	timebfsampling	-0.00 ± 0.00	137	-2.12	**0.03**		
postTRIG							
**logpostTRIG ~ fRC1 + dRC2 + Sex + Year + 1|Band**						0.25	0.74
	Intercept	-0.14 ± 0.10	120	-1.36	0.18		
	fRC1	-0.18 ± 0.05	115	-3.22	**0.002**		
	dRC2	0.12 ± 0.04	89	3.32	**0.001**		
	Sex - M	-0.23 ± 0.08	124	-2.99	**0.003**		
	Year - 2019	0.16 ± 0.07	74.3	2.34	**0.02**		
ΔbCORT							
**ΔbCORT ~ fRC2 + dRC2 + 1|Band**						0.08	0.18
	Intercept	0.36 ± 0.11	117	3.42	**< 0.001**		
	fRC2	-0.29 ± 0.11	117	-2.67	**0.01**		
	dRC2	0.24 ± 0.11	127	-2.19	**0.03**		

Linear mixed model output includes estimates of fixed effects, standard error (SE), degrees of freedom (df), t-statistics (t), p-values (p), marginal R^2 (r_m^2; the proportion of variance in the model explained by the fixed effects) and conditional R^2 (r_c^2; the proportion of variance in the model explained by both fixed and random effects). Significant p-values in bold. See methods for full descriptions of models.

Table S10. Summary of variation in thick-billed murre foraging success – relative change in mass (ΔMass), relative change in triglycerides (ΔTRIG), and nutritional state – post-foraging baseline corticosterone (post-bCORT; log-scaled) from linear models during incubation at Coats Island, Nunavut.

Model	Fixed Effects	Estimate ± SE	t	p	R^2_{adj}
ΔMass					
**ΔMass ~ fRC2 + dRC1* AvgIceConc_day + dRC2 + Year + Duration**					0.33
	Intercept	-0.06 ± 0.02	-2.79	**0.01**	
	fRC2	0.01 ± 0.00	1.50	0.14	
	dRC1	0.00 ± 0.00	0.01	0.99	
	AvgIceConc_day	0.00 ± 0.00	1.50	0.14	
	dRC2	0.02 ± 0.00	4.77	**< 0.001**	
	Year - 2018	0.06 ± 0.02	3.62	**< 0.001**	
	Year - 2019	0.06 ± 0.02	3.74	**< 0.001**	
	Duration	-0.00 ± 0.00	-3.24	**0.001**	
	dRC1*AvgIceConc_day	-0.01 ± 0.00	2.03	**0.04**	
ΔTRIG					
**ΔTRIG ~ fRC1 + fRC2 + dRC2**					0.19
	Intercept	0.14 ± 0.08	1.86	0.07	
	fRC1	-0.32 ± 0.08	-3.93	**< 0.001**	
	fRC2	0.15 ± 0.06	2.70	**0.01**	
	dRC2	0.17 ± 0.04	3.99	**< 0.001**	
post-bCORT					
**logpostbCORT ~ AvgIceConc_day + fRC2 + dRC1**					0.06
	Intercept	0.71 ± 0.23	3.06	**0.003**	
	AvgIceConc_day	0.09 ± 0.06	1.54	0.13	
	fRC2	-0.29 ± 0.10	-2.87	**0.005**	
	dRC1	0.29 ± 0.15	1.98	**0.05**	

Linear model output includes estimates of fixed effects, standard error (SE), t-statistics (t), p-values (p), and adjusted R^2 (R_{adj}^2; the proportion of variance in the model explained fixed effects). Significant p-values bolded. See methods for full descriptions of models.

Table S11. Summary of variation in thick-billed murre nutritional state – post-foraging beta-hydroxybutyrate (post-B-OH; log-scaled), and foraging success – relative change in beta-hydroxybutyrate (ΔB-OH) from linear mixed models during incubation at Coats Island, Nunavut.

Model	Fixed Effects	Estimate ± SE	df	t	p	r_m^2	r_c^2
post-B-OH							
**logpostBOH ~ fRC1 + dRC1 + dRC2 + 1|Band**						0.13	0.74
	Intercept	0.35 ± 0.07	126	5.05	**< 0.001**		
	fRC1	-0.23 ± 0.05	120	-4.27	**< 0.001**		
	dRC1	0.11 ± 0.05	97.2	2.00	**0.05**		
	dRC2	-0.05 ± 0.03	95.9	-1.45	0.15		
ΔB-OH							
**ΔBOH ~ AvgIceConc_day + dRC2 + Year + Duration + 1|Band**						0.12	0.43
	Intercept	-0.05 ± 0.17	127	-0.28	0.78		
	AvgIceConc_day	-0.07 ± 0.03	127	-2.21	**0.03**		
	dRC2	-0.08 ± 0.05	121	-1.79	0.08		
	Year - 2019	0.24 ± 0.09	113	2.81	**0.01**		
	Duration	0.00 ± 0.00	124	1.60	0.11		

Linear mixed model output includes estimates of fixed effects, standard error (SE), degrees of freedom (df), t-statistics (t), p-values (p), marginal R^2 (r_m^2; the proportion of variance in the model explained by the fixed effects) and conditional R^2 (r_c^2; the proportion of variance in the model explained by both fixed and random effects). Significant p-values in bold. See methods for full descriptions of models.

Table S12. Summary of variation in thick-billed murre nutritional state – post-foraging non-esterified fatty acids (NEFA; log-scaled), and foraging success – relative change in non-esterified fatty acids (ΔNEFA) from linear mixed models during incubation at Coats Island, Nunavut.

Model	Fixed Effects	Estimate ± SE	t	p	R^2_{adj}
postNEFA					
**logpostNEFA ~ dRC1 + TimebfSampling**					0.02
	Intercept	-1.31 ± 0.07	-17.5	**< 0.001**	
	dRC1	0.12 ± 0.07	1.62	0.11	
	TimebfSampling	0.00 ± 0.00	1.74	0.08	
ΔNEFA					
**ΔNEFA ~ dRC2 + Year**					0.03
	Intercept	-0.05 ± 0.10	-0.51	0.61	
	dRC2	-0.12 ± 0.07	-1.67	0.10	
	Year - 2019	0.24 ± 0.14	1.75	0.08	

Linear model output includes estimates of fixed effects, standard error (SE), t-statistics (t), p-values (p), and adjusted R^2 (R_{adj}^2; the proportion of variance in the model explained fixed effects). Significant p-values bolded. See methods for full

Table S13. Summary of variation in thick-billed murre foraging behaviour – fRC1 (mean trip distance, mean trip duration, and number of trips per day) and fRC2 (maximum distance, average daily distance, and mean trip distance) and diving behaviour – dRC1 (maximum dive depth, maximum dive duration, mean dive depth, mean dive duration, number of dive bouts per day) from linear models during chick-rearing at Coats Island, Nunavut.

Model	Fixed Effects	Estimate ± SE	df	t	p	r_m^2	r_c^2
**fRC1 ~ Duration + 1|Band**						0.05	0.56
	Intercept	-1.45 ± 0.20	130	-7.18	**< 0.001**		
	Duration	0.01 ± 0.00	115	3.72	**< 0.001**		
**fRC2 ~ Sex + Year + Duration + 1|Band**						0.06	0.42
	Intercept	-0.03 ± 0.31	155	-1.27	0.93		
	Sex - M	-0.29 ± 0.16	104	-1.92	0.07		
	Year - 2018	-0.36 ± 0.16	111	-1.99	**0.03**		
	Year - 2019	-0.39 ± 0.19	145	-2.28	**0.04**		
	Duration	0.01 ± 0.01	153	2.22	**0.05**		
**dRC1 ~ ChickAge + Sex + Year + Duration + 1|Band**						0.10	0.62
	Intercept	0.05 ± 0.32	145	0.17	0.86		
	ChickAge	0.04 ± 0.02	135	2.51	**0.01**		
	Sex - M	-0.33 ± 0.15	109.0	-2.21	**0.03**		
	Year - 2018	0.12 ± 0.14	100	0.80	0.43		
	Year - 2019	-0.36 ± 0.16	124	-2.23	**0.02**		
	Duration	0.01 ± 0.01	133	2.15	**0.03**		

Linear mixed model output includes estimates of fixed effects, standard error (SE), degrees of freedom (df), t-statistics (t), p-values (p), marginal R^2 (r_m^2; the proportion of variance in the model explained by the fixed effects) and conditional R^2 (r_c^2; the proportion of variance in the model explained by both fixed and random effects). Significant p-values in bold. See methods for full descriptions of models.

Table S14. Summary of variation in thick-billed murre diving behaviour – dRC2 (mean dives per bout, number of dive bouts per day, and time diving per day) and average daily energetic expenditure (DEE) from linear mixed models during chick-rearing at Coats Island, Nunavut.

Model	Fixed Effects	Estimate ± SE	df	t	p	r_m^2	r_c^2
**dRC2 ~ AvgSST_day + ChickAge + Sex + 1|Band**						0.29	0.66
	Intercept	-1.07 ± 0.55	149	-1.96	**0.05**		
	AvgSST_day	0.14 ± 0.10	143	1.33	0.19		
	ChickAge	0.01 ± 0.02	138	0.40	0.69		
	Sex - M	1.14 ± 0.16	110	7.02	**< 0.001**		
**DEE ~ fRC1 + fRC2 + dRC2 + Year + 1|Band**						0.85	0.90
	Intercept	2317 ± 29.3	161	79.2	**< 0.001**		
	fRC1	-94.5 ± 18.6	154	-5.08	**< 0.001**		
	fRC2	402 ± 14.6	159	27.5	**< 0.001**		
	dRC2	66.4 ± 13.1	146	5.09	**< 0.001**		
	Year - 2018	-67.2 ± 31.4	113	-2.14	**0.03**		
	Year - 2019	-86.4 ± 29.6	129	-2.92	**0.004**		

Linear mixed model output includes estimates of fixed effects, standard error (SE), degrees of freedom (df), t-statistics (t), p-values (p), marginal R^2 (r_m^2; the proportion of variance in the model explained by the fixed effects) and conditional R^2 (r_c^2; the proportion of variance in the model explained by both fixed and random effects). Significant p-values in bold. See methods for full descriptions of models.

Table S15. Summary of variation in thick-billed murre nutritional state – post-foraging mass (post-Mass) and foraging success – relative change in mass (ΔMass) from linear models during chick-rearing at Coats Island, Nunavut.

Model	Fixed Effects	Estimate ± SE	t	p	R^2_{adj}
postMass					
**postMass ~ fRC2 + dRC1*Sex + ChickAge + TimebfSampling**					0.18
	Intercept	984 ± 13.7	71.8	**< 0.001**	
	fRC2	-18.2 ± 5.70	-3.19	**0.002**	
	dRC1	18.6 ± 7.30	2.55	**0.01**	
	Sex - M	16.2 ± 12.3	1.32	0.19	
	ChickAge	-4.86 ± 1.22	-4.00	**< 0.001**	
	TimebfSampling	-0.58 ± 0.36	-1.6	0.11	
	dRC1*Sex	-24.5 ± 11.8	-2.07	**0.04**	
ΔMass					
**ΔMass ~ AvgSST_day + fRC2 + dRC1 + Sex + Duration**					0.12
	Intercept	0.05 ± 0.03	1.70	0.09	
	AvgSST_day	-0.01 ± 0.00	-2.04	**0.04**	
	fRC2	0.01 ± 0.00	1.66	0.10	
	dRC1	0.01 ± 0.00	1.66	0.10	
	Sex - M	0.02 ± 0.01	2.29	**0.02**	
	Duration	-0.00 ± 0.00	-1.84	0.07	

Linear model output includes estimates of fixed effects, standard error (SE), t-statistics (t), p-values (p), and adjusted R^2 (R_{adj}^2; the proportion of variance in the model explained fixed effects). Significant p-values bolded. See methods for full descriptions of models.

Table S16. Summary of variation in thick-billed murre nutritional state – post-foraging triglycerides (post-TRIG; log-scaled) and foraging success – relative change in triglycerides (Δ TRIG) from linear models during chick-rearing at Coats Island, Nunavut.

Model	Fixed Effects	Estimate ± SE	t	p	R^2_{adj}
post-TRIG					
**logpostTRIG ~ AvgSST_day + fRC2 + Sex**					0.33
	Intercept	1.86 ± 0.71	2.61	**0.01**	
	AvgSST_day	-0.27 ± 0.11	-2.36	**0.02**	
	fRC2	0.16 ± 0.06	2.58	**0.01**	
	Sex - M	-0.41 ± 0.12	-3.37	**0.001**	
ΔTRIG					0.29
**ΔTRIG ~ AvgSST_day + fRC2 + dRC2 + Duration**					
	Intercept	2.67 ± 0.95	2.81	**0.01**	
	AvgSST_day	-0.38 ± 0.12	-3.13	**0.003**	
	fRC2	0.21 ± 0.06	3.66	**0.001**	
	dRC2	0.13 ± 0.05	2.76	**0.01**	
	Duration	-0.01 ± 0.00	-1.61	0.11	

Linear model output includes estimates of fixed effects, standard error (SE), t-statistics (t), p-values (p), and adjusted R^2 (R_{adj}^2; the proportion of variance in the model explained fixed effects). Significant p-values bolded. See methods for full descriptions of models.

Table S17. Summary of variation in thick-billed murre nutritional state – post-foraging baseline corticosterone (post-bCORT; log-scaled) and foraging success – relative change in baseline corticosterone (ΔbCORT) from linear models during the chick-rearing period at Coats Island, Nunavut.

Model	Fixed Effects	Estimate ± SE	t	p	R^2_{adj}
post-bCORT					
**logpostbCORT ~ fRC2 + dRC2*Sex**					0.14
	Intercept	0.75 ± 0.14	5.49	**< 0.001**	
	fRC2	-0.24 ± 0.10	-2.27	**0.03**	
	dRC2	-0.26 ± 0.13	-2.10	**0.04**	
	Sex - M	-0.02 ± 0.27	-0.09	0.93	
	dRC2*Sex	0.52 ± 0.22	2.43	**0.02**	
ΔbCORT					
**ΔbCORT ~ fRC2**					0.03
	Intercept	-0.05 ± 0.14	0.34	0.73	
	fRC2	-0.25 ± 0.14	-1.79	0.08	

Linear model output includes estimates of fixed effects, standard error (SE), t-statistics (t), p-values (p), and adjusted R^2 (R_{adj}^2; the proportion of variance in the model explained fixed effects). Significant p-values bolded. See methods for full

Table S18. Summary of variation in thick-billed murre nutritional state – post-foraging beta-hydroxybutyrate (post-B-OH; log-scaled), relative change in beta-hydroxybutyrate (ΔB-OH), and foraging success – relative change in non-esterified fatty acids (ΔNEFA) from linear models during chick-rearing at Coats Island, Nunavut.

Model	Fixed Effects	Estimate ± SE	t	p	R^2_{adj}
post-B-OH					
**logpostBOH ~ AvgSST_day + dRC1 + ChickAge + Sex**					0.17
	Intercept	-1.06 ± 0.48	-2.23	**0.03**	
	AvgSST_day	0.27 ± 0.08	3.29	**0.002**	
	dRC1	0.11 ± 0.05	2.04	**0.05**	
	ChickAge	-0.02 ± 0.01	-2.07	**0.04**	
	Sex - M	-0.14 ± 0.08	-1.77	0.08	
ΔB-OH					
**ΔBOH ~ AvgSST_day + dRC1*ChickAge**					-0.02
	Intercept	-1.08 ± 0.66	-1.64	0.11	
	AvgSST_day	0.19 ± 0.11	1.67	0.10	
	dRC1	0.38 ± 0.18	2.08	**0.04**	
	ChickAge	-0.01 ± 0.02	-0.30	0.77	
	dRC1*ChickAge	-0.04 ± 0.02	-2.21	**0.03**	
ΔNEFA					
**ΔNEFA ~ Sex**					0.07
	Intercept	0.13 ± 0.10	1.36	0.18	
	Sex - M	-0.35 ± 0.15	-2.38	**0.02**	

Linear model output includes estimates of fixed effects, standard error (SE), t-statistics (t), p-values (p), and adjusted R^2 (R_{adj}^2; the proportion of variance in the model explained fixed effects). Significant p-values bolded. See methods for full descriptions of models.

Table S19. Summary of variation in thick-billed murre nutritional state – post-foraging non-esterified fatty acids (post-NEFA; log-scaled) from linear mixed models during chick-rearing at Coats Island, Nunavut.

Model	Fixed Effects	Estimate ± SE	df	t	p	r_m^2	r_c^2
post-NEFA							
**logpostNEFA ~ fRC1 + Sex + 1|Band**						0.15	0.51
	Intercept	-0.96 ± 0.09	57.8	-10.1	**< 0.001**		
	fRC1	-0.17 ± 0.08	64.2	-2.05	**0.04**		
	Sex - M	-0.35 ± 0.13	60.9	-2.75	**0.01**		

Linear mixed model output includes estimates of fixed effects, standard error (SE), degrees of freedom (df), t-statistics (t), p-values (p), marginal R^2 (r_m^2; the proportion of variance in the model explained by the fixed effects) and conditional R^2 (r_c^2; the proportion of variance in the model explained by both fixed and random effects). Significant p-values in bold. See methods for full descriptions of models.
